# High ultrafiltration rate induced intradialytic hypotension is a predictor for cardiac remodeling: a 5-year cohort study

**DOI:** 10.1080/0886022X.2020.1853570

**Published:** 2020-12-13

**Authors:** Jinbo Yu, Xiaohong Chen, Yang Li, Yaqiong Wang, Zhonghua Liu, Bo Shen, Jie Teng, Jianzhou Zou, Xiaoqiang Ding

**Affiliations:** a Division of Nephrology, Zhongshan Hospital, Shanghai Medical College, Fudan University, Shanghai, P. R. China; b Shanghai Institute of Kidney Disease and Dialysis, Shanghai, P. R. China; c Shanghai Key Laboratory of Kidney and Blood Purification, Shanghai, P. R. China

**Keywords:** Intradialytic hypotension, cardiac remodeling, ultrafiltration rate, hemodialysis, left ventricular mass index

## Abstract

**Background:**

Intradialytic-hypotension (IDH) is a common complication of hemodialysis. High ultrafiltration rate (UFR) might lead to IDH. However, the relationships between UFR, IDH, and cardiac remodeling among hemodialysis patients in the long-term have not been deeply explored.

**Methods:**

This retrospective cohort study collected clinical and echocardiographic data. Patients were enrolled from 1 January 2014 to 31 March 2014 and were followed-up for 5-year. Those who suffered from more than four hypotensive events during three months (10% of dialysis treatments) were defined as the IDH group. Subgroup analysis was done according to the UFR of 10 ml/h/kg. Associations between UFR, IDH, and alterations of cardiac structure/function were analyzed.

**Results:**

Among 209 patients, 96 were identified with IDH (45.9%). The survival rate of IDH patients was lower than that of no-IDH patients (65.5% vs. 81.4%, *p* = .005). In IDH group, decreased ejection fraction (EF), larger left atrium diameter index (LADI), and left ventricular mass index (LVMI) (*p* < .05) were observed at the end of the follow-up. In multivariate logistic model, the interaction between UFR and IDH was notably associated with LVMI variation (*OR* = 1.37). After adjusting covariates, UFR was still an independent risk factor of LVMI variation (*OR* = 1.52) in IDH group. In subsequent analysis, we divided patients according to UFR 10 ml/h/kg. For IDH-prone patients, decreased EF, larger LADI, and LVMI (*p* < .05) were observed at the end of the study only in high-UFR group.

**Conclusions:**

UFR and IDH have interactions on cardiac remodeling. High ultrafiltration rate induced IDH is a predictor for cardiac remodeling in long-term follow-up.

## Introduction

Intradialytic-hypotension (IDH) is reported to be one of the most common complications of hemodialysis (HD), occurring in between 10 and 70% of HD sessions depending on different definitions used [1–3]. Many studies have shown that IDH may be a potential factor for poor prognosis. For the short-term, IDH might result in discomfort and inadequate dialysis, contributing to an early end of the dialysis session. While for the long-term, IDH could lead to cardiovascular complications, more hospitalization, and all-cause mortality [4,5]. The possible mechanisms of IDH are excessive and rapid ultrafiltration and loss of cardiac compensatory response. Over ultrafiltration brings about the decrease of arterial blood volume, the decrease of cardiac filling and cardiac output, and finally leads to hypotension. Thus, IDH itself could suggest hemodynamic instability. Studies have shown that cardiac structural alterations have occurred at the early stage of renal insufficiency [6,7], and the alterations become worse after dialysis initiation [8]. The injuries of cardiovascular illnesses, whether present before or after maintenance dialysis initiation, and compensatory mechanisms, could trigger the cardiac remodeling process. Furthermore, cardiac remodeling could contribute to hemodynamic instability. Over ultrafiltration leads to hemodynamic instability as well. So cardiac remodeling and excessive ultrafiltration might be important determinants to IDH.

However, the relationship between IDH and cardiac remodeling has not yet been deeply explored. We presume that ultrafiltration rate (UFR), IDH, and cardiac remodeling might have interaction. Our study aimed to assess the associations between UFR, IDH, and cardiac structural/functional alterations among maintenance hemodialysis (MHD) patients in long-term follow-up.

## Materials and methods

### Patients

End-stage kidney disease patients who started HD before January 2014 in blood purification center, Zhongshan Hospital, Fudan University, Shanghai, China, were enrolled. Inclusion criteria were: ① Patients older than eighteen, ② undergoing dialysis three times a week, ③ without cardiac dysfunction, ④ on HD for more than three months with acceptable dialysis efficiency. They were followed until 31 December 2019. Dry weight was monitored and reevaluated every month by designated physicians according to physical examinational findings, and NT-proBNP measured monthly [9–11], to achieve an edema-free state.

### IDH definition and patient group

Epidemiologic studies about IDH with outcomes are based merely on BP values [1]. But the IDH definition of nadir BP might exclude the effects of patients’ predialysis BP and volume status, which are the risk factors of IDH and very important to patients’ prognosis. So, the definition of IDH we used in our study was defined as a sudden drop in systolic BP more than 20 mmHg or mean artery pressure (MAP) more than 10 mmHg associated with clinical events and the need for interventions [2,3]. The intervention of IDH depends on the rapid identification and intervention of nurses. Typical interventions include repositioning the patient to improve hemoperfusion of vital organs (supine or Trendelenburg posture), cessation of ultrafiltration, and fluid resuscitation with isotonic, hypertonic, or glucose solutions, and adjust dialysate temperature, as well as cessation of dialysis [12,13]. Blood pressures (BPs) were the average values of all the BPs taken hourly intradialysis during the three-month recruitment phase.

Patients who suffered from more than four hypotensive events in three months (10% of HD treatments) were defined as the IDH group [1,5,14,15]. Moreover, subgroups were defined according to the normalized ultrafiltration rate of 10 mL/h/kg [15–17].

### Date collection and biochemical measurements

Patients’ attending physicians extracted information like demographic data, comorbidity, biochemistry, and antihypertensive drugs, from charts at the beginning of the recruitment period and the end of the study. In the morning of 8–10 midweek non-dialysis day, blood samples were taken after 30 min of rest in the semi- reclining position.

### Endpoint events

The primary objective was to identify the association between IDH and the alterations in cardiac structure and function. The second objective was to evaluate the effect of UFR on cardiac structural and functional alterations.

The alterations in cardiac structure and function were measured by echocardiography. An echocardiographic machine (Philips IE33, Eindhoven, The Netherlands) with a 3.5-MHz multiphase array probe by a single experienced cardiologist was used during a midweek non-dialysis day, and transthoracic echocardiographic examinations were conducted within two hours after blood sampling. The echocardiography was done both at the run-in period and the end of the five-year follow-up. Cardiac structure was measured at the end- diastolic phase according to the recommendations of the Penn Convention. The left ventricular ejection fraction (LVEF), used to evaluate the cardiac function, was determined by two-dimensional echocardiography. The left atrium diameter index (LADI) was calculated by dividing the left atrium diastolic diameter (LADD) by body surface area (BSA). The Devereux formula was used to calculate the LV mass. Left ventricular mass index (LVMI) was obtained by dividing LV mass by height in meters rose to the power of 2.7. Left ventricular hypertrophy (LVH) was defined as LV mass/height^2.7^ ≥ 47 g/m^2.7^ in women and ≥ 50/m^2.7^ in men. LVMI variation was defined as the absolute percentage difference of LVMI between the baseline and the end of the study. The variation of LVMI over 50% was considered significant in the clinical base. Relative wall thickness (RWT) was calculated as two × posterior wall thickness/LV internal linear dimension in diastole. Based on the LVMI and RWT measurements [18], four geometric patterns were described: ① normal (normal LVMI and normal RWT), ② concentric remodeling (normal LVMI and increased RWT), ③ eccentric hypertrophy (abnormally increased LVMI and normal RWT), ④ concentric hypertrophy (abnormally increased LVMI and increased RWT).

### Statistical analysis

Continuous variables were presented as mean ± Standard Deviation, while categorical variables were expressed as numbers and percentages appropriately. Student’s *t*-test was used to compare normal variables, whereas for categorical variables, chi-square tests were performed, respectively. Survival probability and median survival time are used to illustrate mortality in IDH and no-IDH groups. The predictive effect of ultrafiltration on the onset of IDH was analyzed using univariate and multivariate logistic regression models. A series of models were conducted: (1) Model 1: adjusted for demographic data (age, sex, and body mass index); (2) Model 2: adjusted for model 1 + dialysis information (interdialytic weight gain, residual renal function, dialysis vintage and single-pool Kt/V); (3) Model 3: adjusted for model 2 + comorbid conditions (history of primary hypertension, coronary heart disease, and diabetes); (4) Model 4: adjusted for model 3 + biochemical data (serum albumin, pre-albumin, creatinine and hemoglobin); (5) Model 5: adjusted for model 4 + cardiac conditions (NT- proBNP, LV mass index, LVEF, and left atrium diameter index); (6) Model 6: adjusted for model 5+ predialysis BP (predialysis systolic BP and diastolic BP). Paired *t*-test was used to evaluate the cardiac alteration cardiac structure (LADI; RWT; LVMI) and cardiac function (LVEF) both at the beginning and the end of the study in different subgroups. We used univariate and multivariate logistic regression models to explore the interaction between UFR and IDH on LVMI variation. Covariates with *p*-values less than .05 in the univariate analysis were included in the multivariate models. All calculations were performed using SPSS version 24 (SPSS Inc., Chicago, IL). *p* < .05 was considered statistically significant.

## Results

### Patient characteristics and the incidence of IDH

A total of 209 patients (118 males and 91 females) with an average age of 52.92 ± 18.68 (18–75) years were collected. 96 cases of IDH (≥ 4 hypotension events/3 months) and 113 cases without IDH (< 4 hypotension events/3 months). Table 1 showed demographic, clinical, and biochemical variables. IDH-prone patients were older, had higher BMI, interdialytic weight gain (IDWG) and ultrafiltration rate, lower predialysis and post-dialysis BP (*p* < .05). Compared with those with no-IDH, there were more female patients and fewer patients with preserved residual kidney function in IDH group (*p* < .01). There were no significant differences found in both the dosage and types of antihypertensive medications, as well as comorbidities (e.g., cardiovascular disease and diabetes) between the two groups (*p* > .05).

**Table 1.** Demographic, clinical and biochemical data of the patients.

Characteristics	Total (*n* = 209)	no-IDH (*n* = 113)	IDH (*n* = 96)	*p* value
Age, years	56.06 ± 13.87	52.63 ± 13.42	60.29 ± 13.31	<.001
Male	118 (60.2%)	81 (71.7%)	37 (44.6%)	<.001
Normalized ultrafiltration rate, ml/h/kg	10.69 ± 4.14	9.65 ± 4.33	11.97 ± 3.50	<.001
IDWG, kg	3.85 ± 1.58	3.43 ± 1.65	4.35 ± 1.33	<.001
Preserved residual kidney function	41 (20.9%)	35 (31%)	6 (7.2%)	<.001
Duration of dialysis, months	30.57 ± 38.25	30.69 ± 41.11	30.41 ± 34.20	.953
Dry weight, kg	58.67 ± 10.81	59.44 ± 10.16	57.73 ± 11.56	.259
spKt/Vurea	1.53 ± 0.63	1.57 ± 0.71	1.48 ± 0.53	.405
BMI, kg/m^2^	22.32 ± 4.01	21.67 ± 3.68	23.20 ± 4.29	.008
Serum albumin, g/L	39.14 ± 3.54	38.90 ± 3.78	39.42 ± 3.25	.342
Serum creatinine, μmol/L	995.30 ± 286.09	974.28 ± 298.93	1022.81 ± 267.71	.251
Hemoglobin, g/L	102 ± 25.24	99.28 ± 29.06	105.70 ± 18.39	.745
NT-proBNP, pg/mL	5051.94 ± 5472.11	4660.82 ± 5266.30	5547.72 ± 5721.52	.309
iPTH, ng/L	435.05 ± 462.15	393.52 ± 375.44	486.04 ± 548.57	.187
Serum β_2_MG, mg/L	29.36 ± 12.59	30.58 ± 11.96	27.88 ± 13.24	.176
hs CRP, mg/L	7.15 ± 12.26	7.62 ± 13.22	6.62 ± 11.15	.590
Predialysis-SBP, mmHg	136.63 ± 17.67	139.27 ± 13.19	133.52 ± 21.46	.019
Predialysis-DBP, mmHg	82.44 ± 10.81	84.47 ± 9.21	80.06 ± 12.05	.003
Postdialysis-SBP, mmHg	125.46 ± 19.26	133.3 ± 15.9	116.14 ± 18.81	<.001
Postdialysis-DBP, mmHg	78.45 ± 10.44	82.46 ± 8.37	73.68 ± 10.69	<.001
EF, %	63.18 ± 9.25	63.22 ± 7.69	63.13 ± 11.47	.962
LADI, mm/m^2^	24.91 ± 4.17	24.55 ± 4.32	25.49 ± 3.91	.238
RWT	0.46 ± 0.12	0.44 ± 0.80	0.49 ± 0.16	.057
LVMI, g/m^2.7^	47.33 ± 18.62	47.67 ± 16.07	46.92 ± 21.38	.798
Antihypertensive medication dosage, tablets	2.39 ± 1.80	2.32 ± 1.80	2.54 ± 1.84	.612
Antihypertensive medication type	1.98 ± 1.34	1.95 ± 1.34	2.04 ± 1.34	.778
Hypertension	129 (65.8%)	75 (66.4%)	54 (65.1%)	.849
Diabetes mellitus	31 (15.8%)	15 (13.3%)	16 (19.3%)	.257
Cardiovascular disease	18 (9.2%)	12 (10.6%)	6 (7.2%)	.419
Cerebrovascular disease	3 (1.5%)	1 (0.9%)	2 (2.4%)	.393

Values are mean (SD) for continuous variables and % (n) for categorical variables.

IDWG: interdialytic weight gain; BMI: body mass index; NT-proBNP: N-terminal pro-B-type natriuretic peptide; iPTH: intact parathyroid hormone; β_2_MG: β_2_-microglobulin; hs-CRP: high-sensitive c-reactive protein; EF: ejection fraction; LADI: left atrium diameter index; RWT: relative wall thickness; LVMI: left ventricular mass index; Preserved residual kidney function: 24 h urine output over 100 mL.

### IDH and prognosis

**Figure 1.**
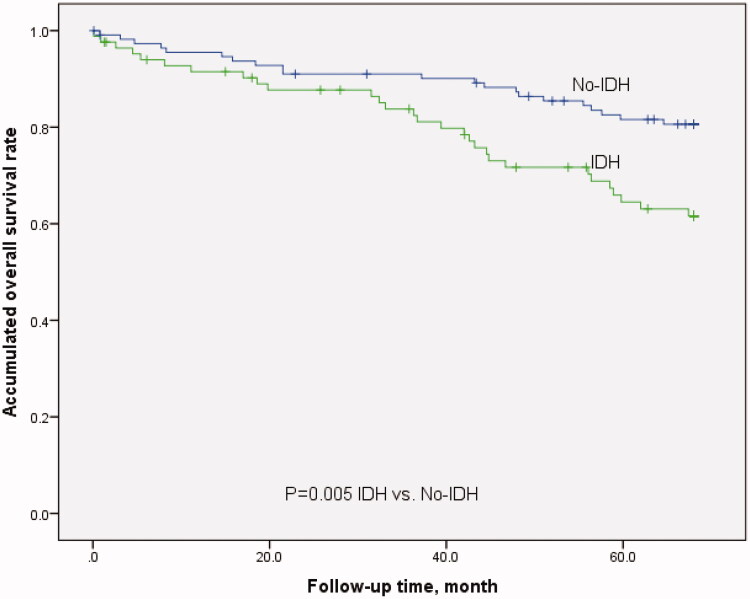
Kaplan–Meier survival curves of patients with IDH and no-IDH.

Among 209 patients, 12 (5.7%) were excluded due to kidney transplantation and transfer to other HD centers during the five-year observation period. The mortality rate was 5.1/100-person-year, including 15 cardiovascular events, 13 cerebrovascular events, nine severe infection deaths, eight sudden deaths, and five cancer deaths. The survival rate of IDH patients was lower than that of patients with no-IDH (shown in Figure 1: 65.5% vs. 81.4%, *p* = .005).

### UFR and IDH

We constructed a series of logistic regression models to test the predictive effects of UFR on the onset of IDH. After adjusting for confounding factors like demographic data (age, sex, and body mass index), dialysis information (interdialytic weight gain, residual renal function, dialysis vintage and single-pool Kt/V), comorbid conditions (history of primary hypertension, coronary heart disease, and diabetes), biochemical data (serum albumin, pre-albumin, creatinine and hemoglobin), cardiac conditions (NT- proBNP, LV mass index, LVEF, and left atrium diameter index), and predialysis BP (predialysis systolic BP and diastolic BP), UFR was still a predictor for IDH (*p* < .05) (Table 2).

**Table 2.** Logistic regression models of UFR on IDH.

Model	HR	95%CI	*p* value
Ultrafiltration (continuous variable, per 1ml/h/kg increase)			
Unadjusted	1.16	1.08–1.26	<.001
Model 1	1.24	1.12–1.39	<.001
Model 2	1.20	1.03–1.40	.017
Model 3	1.20	1.04–1.40	.016
Model 4	1.22	1.00–1.47	.042
Model 5	1.65	1.05–2.61	.031
Model 6	2.37	1.21–4.62	.012

UFR: ultrafiltration rate; IDH: intradialytic-hypotension.

Model 1: adjusted for demographic data (age, sex, and body mass index).

Model 2: adjusted for Model 1 + dialysis information (interdialytic weight gain, residual renal function, dialysis vintage and single-pool Kt/V).

Model 3: adjusted for Model 2 + comorbid conditions (history of primary hypertension, coronary heart disease, and diabetes).

Model 4: adjusted for Model 3 + biochemical data (serum albumin, pre-albumin, creatinine and hemoglobin).

Model 5: adjusted for Model 4 + cardiac conditions (NT- proBNP, left ventricular mass index, left ventricular ejection fraction, and left atrium diameter index).

Model 6: adjusted for Model 5+ predialysis blood pressure (predialysis systolic blood pressure and diastolic blood pressure).

### IDH and cardiac structural/functional alterations

We compared cardiac structure and function at the recruitment time with those at the end of the 5-year study by echocardiography. In the IDH group, longer LV end-diastolic diameter, decreased LVEF, larger LADI as well as larger LVMI (*p* < .05, *p* < .01) were observed at the end of the follow-up than at the recruitment time (Table 3). However, similar results were not found in the no-IDH group.

**Table 3.** IDH and cardiac structural/ functional alterations.

Echocardiography parameters	Total			no-IDH			IDH		
	Mean ± SD		*p* value	Mean ± SD		*p* value	Mean ± SD		*p* value
	recruitment period	end of the study		recruitment period	end of the study		recruitment period	end of the study	
AORD, mm	32.71 ± 3.27	33.61 ± 5.07	.119	33.66 ± 3.37	34.36 ± 3.79	.271	31.45 ± 2.69	32.62 ± 6.30	.272
LAD, mm	39.95 ± 5.44	40.59 ± 7.61	.516	40.30 ± 6.39	41.27 ± 6.96	.446	39.48 ± 3.87	39.69 ± 8.40	.894
LVDD, mm	46.00 ± 6.52	50.37 ± 7.14	<.001	47.26 ± 5.94	50.59 ± 7.51	.013	44.30 ± 6.95	50.07 ± 6.68	.001
LVDS, mm	30.29 ± 5.38	31.36 ± 6.84	.256	31.20 ± 5.99	31.45 ± 6.00	.843	29.05 ± 4.19	31.24 ± 7.92	.127
IVST, mm	10.71 ± 1.83	11.00 ± 2.64	.376	10.72 ± 1.92	10.74 ± 2.06	.962	10.70 ± 1.74	11.35 ± 3.26	.272
LVPWT, mm	9.96 ± 1.51	10.53 ± 1.87	.018	9.98 ± 1.58	10.41 ± 1.71	.143	9.93 ± 1.42	10.70 ± 2.09	.062
LVEF, %	65.95 ± 7.41	63.89 ± 6.45	.068	65.47 ± 7.81	64.00 ± 6.82	.336	67.39 ± 5.72	63.55 ± 5.88	.010
LADI, mm/m^2^	24.53 ± 4.10	25.38 ± 4.70	.135	24.64 ± 4.13	24.26 ± 4.43	.623	24.74 ± 3.58	26.40 ± 5.32	.015
RWT	0.43 ± 0.09	0.45 ± 0.08	.128	0.43 ± 0.09	0.44 ± 0.08	.312	0.44 ± 0.10	0.47 ± 0.09	.257
LVMI, g/m^2.7^	45.21 ± 11.77	53.15 ± 19.89	.001	45.50 ± 11.88	49.28 ± 16.86	.173	44.77 ± 11.74	58.96 ± 22.75	<.001

Values are mean (SD) for continuous variables.

AoRD: aortic root inside diameter; LAD: left atrium diameter; LVDD: left ventricular end-diastolic diameter; LVDS: left ventricular end-systolic dimension; IVST: interventricular septal thickness; LVPWT: left ventricular posterior wall thickness; LVEF: left ventricular ejection fraction; LADI: left atrium diameter index; RWT: relative wall thickness; LVMI: left ventricular mass index.

### Interactions between UFR and IDH on cardiac remodeling

As UFR is a risk factor of IDH, they might have interactive effects on LVMI variation. We presumed that UFR might have different prognostic effects on LVMI variation in IDH and no-IDH groups. We divided all of the patients into two groups according to LVMI variations of 50% and constructed logistic regression models to test the interaction effects of UFR and IDH on LVMI variations. Crude model 1 showed that the interaction of IDH and UFR had a significant association with LVMI variation (*p* = .010) (Table 4). Subgroup analysis showed that in IDH group, UFR was an independent risk factor of LVMI variation (*OR* = 1.26, 95%CI 1.05–1.50), while in the no-IDH group, such result was not found (*OR* = 0.95, 95%CI 0.84–1.07). Besides, we created a multivariate logistic regression model consisting of demographic data (age, gender, and BMI) and cardiac conditions (cardiac comorbidity and ejection fraction), the interaction between UFR and IDH was notably associated with LVMI variation (*p* = .014). After adjusting these covariates in subgroups, UFR was still an independent risk factor of LVMI variation (*OR* = 1.52, 95%CI 1.17–1.98) in the IDH group, while there were no such findings (*OR* = 0.95, 95%CI 0.81–1.10) in the no-IDH group.

**Table 4.** Interactions between UFR and IDH on LVMI variation.

		*OR*	*p* value
Crude model 1	IDH	0.10 (0.01 ∼ 1.32)	.080
	UFR, ml/h/kg	0.95 (0.84 ∼ 1.07)	.364
	IDH × UFR, ml/h/kg	1.33 (1.07 ∼ 1.65)	.010
Adjusted model 2	IDH	0.07 (0.00 ∼ 1.28)	.072
	UFR, ml/h/kg	0.99 (0.85 ∼ 1.15)	.875
	IDH × UFR, ml/h/kg	1.37 (1.07 ∼ 1.77)	.014

IDH: intradialytic hypotension; UFR: ultrafiltration rate; LVMI: left ventricular mass index model 2: adjusted for age, gender, body mass index, cardiovascular comorbidity (coronary heart disease and arrhythmia), ejection fraction.

Furthermore, we divided IDH-prone patients into two groups according to UFR 10 mL/h/kg [15–17]. We compared cardiac structure and function at the recruitment time with those at the end of the study in both IDH-prone groups. During the 5-year follow-up, in the high-UFR group, longer aortic root inside diameter, LV end-diastolic diameter, LV end-systolic dimension, and decreased EF, large LADI, as well as larger LVMI (*p* < .05) were observed at the end of the follow-up. While there were no significant alterations in cardiac structure and function in the low-UFR group throughout the study. In the no-IDH group, no significant alterations were observed in cardiac structure and function with different levels of UFR (Table 5). For patients without primary cardiac disease, abnormally increased LVMI, RWT and LADI were found in IDH patients. Similar results were found in IDH patients with high-UFR (Supplemental data). High ultrafiltration rate induced IDH is a determinant of cardiac remodeling.

**Table 5.** UFR and cardiac structural/ functional alterations in both groups.

Echocardiography parameters	IDH group						no-IDH group					
	High-UFR			Low-UFR			High-UFR			Low-UFR		
	Mean ± SD		*p*-value	Mean ± SD		*p*-value	Mean ± SD		*p*-value	Mean ± SD		*p*-value
	recruitment period	end of the study		recruitment period	end of the study		recruitment period	end of the study		recruitment period	end of the study	
AORD, mm	31.00 ± 2.52	33.36 ± 3.62	.001	33.11 ± 2.76	29.89 ± 11.84	.453	33.54 ± 2.48	33.58 ± 4.77	.968	33.75 ± 3.94	34.94 ± 2.79	.135
LAD, mm	39.30 ± 3.70	41.12 ± 6.03	.167	40.11 ± 4.62	34.44 ± 13.31	.338	41.46 ± 7.53	41.33 ± 8.02	.956	39.44 ± 5.34	41.22 ± 6.19	.219
LVDD, mm	44.26 ± 6.07	50.88 ± 6.71	<.001	44.44 ± 10.08	47.00 ± 5.92	.575	47.40 ± 6.68	50.96 ± 8.86	.152	47.15 ± 5.42	50.30 ± 6.44	.034
LVDS, mm	29.13 ± 3.18	32.22 ± 6.41	.027	28.78 ± 6.96	27.78 ± 11.72	.825	31.96 ± 6.96	31.25 ± 7.18	.764	30.63 ± 5.18	31.59 ± 5.06	.476
IVST, mm	10.59 ± 1.79	11.50 ± 3.47	.205	11.11 ± 1.54	10.78 ± 2.39	.700	11.48 ± 2.10	10.32 ± 2.12	.068	10.15 ± 1.56	11.06 ± 1.98	.02
LVPWT, mm	10.00 ± 1.37	10.74 ± 1.97	.098	9.67 ± 1.66	10.56 ± 2.60	.426	10.52 ± 1.85	9.92 ± 1.66	.206	9.58 ± 1.23	10.79 ± 1.67	.001
EF, %	68.08 ± 5.84	63.28 ± 6.26	.010	65.25 ± 5.09	64.38 ± 4.81	.667	65.00 ± 9.66	63.16 ± 6.90	.52	65.82 ± 6.18	64.64 ± 6.80	.469
LADI, mm/m^2^	25.28 ± 3.76	27.50 ± 5.43	.040	24.35 ± 3.75	24.36 ± 4.54	.978	25.14 ± 4.66	25.22 ± 5.27	.958	23.55 ± 3.62	24.26 ± 3.72	.398
RWT	0.43 ± 0.10	0.46 ± 0.08	.283	0.46 ± 0.10	0.49 ± 0.11	.659	0.41 ± 0.09	0.47 ± 0.09	.027	0.42 ± 0.05	0.44 ± 0.09	.285
LVMI, g/m^2.7^	45.87 ± 11.03	63.67 ± 22.75	<.001	40.62 ± 14.16	41.30 ± 11.89	.889	45.96 ± 12.00	46.93 ± 19.11	.849	45.16 ± 11.97	50.99 ± 15.09	.063

Values are mean (SD) for continuous variables.

LVEF: left ventricular ejection fraction; LADI: left atrium diameter index; RWT: relative wall thickness; LVMI: left ventricular mass index; AoRD: aortic root inside diameter; LAD: left atrium diameter; LVDD: left ventricular end-diastolic diameter; LVDS: left ventricular end-systolic dimension; IVST: interventricular septal thickness; LVPWT: left ventricular posterior wall thickness.

## Discussion

In order to explore the interactive effect of UFR and IDH on cardiac remodeling, we conducted this retrospective cohort study. We found that the interaction between UFR and IDH could predict cardiac remodeling in long-term follow-up. This finding was independent of confounding risk factors. Furthermore, we found that for IDH-prone patients, high UFR would lead to cardiac remodeling in long-term follow-up.

Cardiac remodeling found at the initiation of dialysis may cause hemodynamic instability. In addition, the sequelae of cardiac remodeling may be a positive factor of dialysis complications, especially IDH [19]. To some extent, LVH lays the foundation for IDH occurrence through LV stiffening [20] as well as myoischemia and arrhythmia [21]. However, few researchers have explored IDH and its effect on cardiac remodeling. In this study, we focused on the potential contribution of IDH to the development of cardiac remodeling.

It is commonly recognized that the process of HD itself causes myocardial ischemia. IDH refers to intradialytic hemodynamic instability, linked to episodic myocardial stunning [22,23]. Repeated cardiac ischemia can result in myocardial hypertrophy and fibrosis, reduce the response to filling pressure, and increase the risk of hemodynamic compromise. Endothelial dysfunction, impaired calcium regulation, and reperfusion after IDH (the production of free radicals in the myocardium) bring about this phenomenon [24]. Myocardial stunning may cause LV dysfunction and structure alteration, leading to heart failure. In our study, we found no significant difference of LVEF in both groups of patients at baseline, while decreased LVEF was observed in the IDH group in long-term follow-up (*p* = .010). Repetitive ischemia episodes are cumulative, contributing to cardiac fibrosis and cardiac hypertrophy, causing systolic LV dysfunction and LVH. So IDH could cause cardiac dysfunction in the long term.

Intermittent volume retention caused by IDH makes patients suffer from chronic volume overload and, eventually leads to myocardial remodeling. When BP drops, LV diastolic function is crucial to sustain adequate cardiac filling. LVH and significant diastolic dysfunction are common in HD patients, and they bear the burden of decreased myocardial compliance secondary to increased fibrosis. The resulting increase in LV stiffness resists chamber filling. In this case, the LV volume filling pressure curve is steep, and the rapid reduction of LV volume will lead to a low output state, such as IDH. The left atrium (LA) is considered to maintain LV diastole pressures, so LA structures and functions are often marked as indirect indicators for diastolic function [25]. The left atrium dimension index (LADI) is suggested to be used to evaluate the LV diastolic function [26]. Previous studies [27,28] revealed that left atrium enlargement could predict IDH. Volume-dependent (preload) factors, such as salt and volume overload, lead to cardiomyocyte lengthening and eccentric remodeling. Stress-dependent (afterload) factors such as aortic stiffness and hypertension predisposition toward cell thickening and concentric remodeling. On the contrary, those with eccentric hypertrophy are accompanied by coronary artery diseases, leading to LV dilatation and systolic dysfunction in the long-term [29]. Eccentric LV hypertrophy is related to mean peripheral resistance, but a high cardiac index is consistent with excessive circulating blood volume [18]. So LVH sets the background for IDH development. In our study, LADI and LVMI were used to evaluate left atrial enlargement and LVH. We found that the frequent onset of IDH might predict cardiac remodeling, including LA dilatation, eccentric LV hypertrophy, and LVH, in long-term follow-up.

IDH occurs when dialysis ultrafiltration exceeds the plasma reperfusion rate of standard physiologic compensatory mechanisms. UFR and total volume removal are risk factors of IDH [13,14]. Nevertheless, we also found that UFR was an independent risk factor for IDH in a series of models. Studies suggested that UFR >10 mL/h/kg was related to a higher risk of mortality and a higher risk of IDH [16,17,30]. So, we use UFR 10 mL/h/kg to evaluate the impact of different volume status on cardiac alterations. We found that in the IDH group with high UFR, decreased LVEF, larger LADI, and LVMI were shown at the end of the study. So high UFR would cause cardiac remodeling only in IDH-prone patients.

IDH might cause coronary hypoperfusion, myocardial stunning [22], and RAAS dysregulation [31–33], contributing to LVH. This study divided all of the patients into two groups according to the LVMI variation of 50% and constructed logistic regression models to test the interaction effects of UFR and IDH on LVMI variations. Both univariate and multivariate regression models suggested that the interaction between UFR and IDH was notably associated with LVMI variation. Subgroup analysis showed that after adjusting for the demographic data (age, gender, and BMI) and cardiac conditions (cardiac comorbidity and ejection fraction), UFR was still an independent risk factor of variation of LVMI (*OR* = 1.52) in the IDH group. At the same time, there were no such findings (*OR* = 0.95) in the no-IDH group. Thus, we presume that long-term over ultrafiltration for some of the IDH-prone patients infers cardiac overload, leading to cardiac dysfunction and LVH in long-term follow-up. IDH and over ultrafiltration might have a synergistic effect on cardiac remodeling.

This study comprehensively assesses the risk factors (including UFR and IDH) for cardiac remodeling in MHD patients. We firstly testified the contribution of IDH to cardiac remodeling in the clinical base. Another is the consideration of ultrafiltration, which is relevant both to IDH and cardiac remodeling. Furthermore, we have a long-term follow-up period.

However, our study has several limitations. First, the sample size was rather small. The results might not be validated in other populations. Nevertheless, patients of the study were free of cardiac dysfunction, and we did not have enough information on diastolic function (e.g., e/e’ or E/A). Further research might target whether our study results could be testified in MHD patients with various comorbidities. An additional limitation is that the effect of varied vascular accesses on hemodynamic stability was not assessed. Fourth, we did not use objective methods (e.g., bio-impedance) to assess fluid status, which is more objective and accurate to determine volume status. The last important limitation is that we did not have information about specific interventions and adjustments of antihypertensive medications (e.g., angiotensin converting enzyme inhibitors and β-receptor blockers) that may confound and modify our associations.

## Conclusions

IDH and myocardial remodeling, especially LVH, are closely related to each other. On the one hand, LVH is an important determinant and etiology of IDH, because LVH paves the way for the frequent occurrence of IDH, and actively promotes the BP drop during dialysis through mechanisms including arrhythmia and myocardial ischemia. On the other hand, IDH might predict cardiac remodeling due to long-term over-ultrafiltration. Excessive ultrafiltration might not induce cardiac remodeling in patients with better compensatory mechanisms. However, over ultrafiltration for IDH-prone patients causes decreased cardiac output in the long-term, contributing to cardiac dysfunction and remodeling. Strategies, including proper ultrafiltration setting, should be taken to maintain hemodynamic stability, especially in IDH-prone patients.

## Supplementary Material

Supplemental Material
