# Supplementary figures and images for: High ultrafiltration rate induced intradialytic hypotension is a predictor for cardiac remodeling: a 5-year cohort study
Source: Ren Fail. 2020 Dec 13;43(1):40–8. doi: 10.1080/0886022X.2020.1853570 (PMC7745843; doi:10.1080/0886022X.2020.1853570)

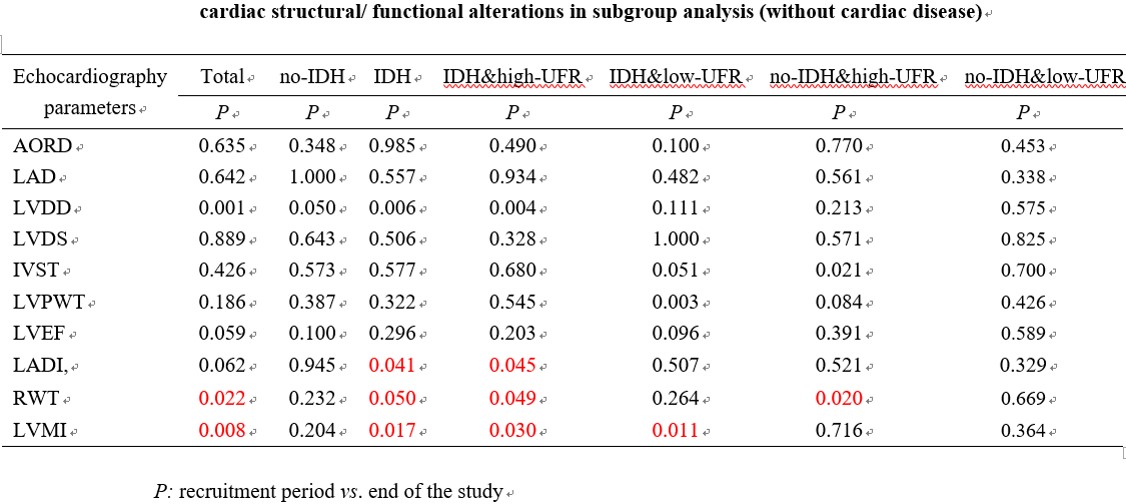

Supplement: Supplemental Material [file IRNF_A_1853570_SM5581.jpg]
